# Narrative participation as a context for meaning-making in early school years: a mixed-methods study of emotion comprehension

**DOI:** 10.3389/fpsyg.2026.1833673

**Published:** 2026-06-03

**Authors:** Orsolya Nemet, Szilvia Fodor, Klára Soltész-Várhelyi, Jozsef Racz, Aniko Zsolnai

**Affiliations:** 1 Doctoral School of Psychology, Eötvös Loránd University (ELTE), Budapest, Hungary; 2 Department of School Psychology and Educational Psychology, Eötvös Loránd University, Institute of Psychology, Budapest, Hungary; 3 Institute of Psychology, Pázmány Péter Catholic University, Budapest, Hungary; 4 Department of Psychological Counseling and Therapeutic Interventions, Eötvös Loránd University, Institute of Psychology, Budapest, Hungary; 5 Department of Addictology, Faculty of Health Sciences, Semmelweis University, Budapest, Hungary; 6 Eötvös Loránd University, Institute of Education, Budapest, Hungary

**Keywords:** early elementary education, emotion comprehension, meaning-making, narrative participation, social–emotional learning (SEL)

## Abstract

Emotion comprehension develops through children’s engagement with emotionally and socially meaningful experiences, yet less is known about how narrative-based classroom engagement may support this process. The T. A. L. E. (Tale Activates Learning and Emotional-Growth) intervention, developed by the authors, uses culturally diverse oral storytelling embedded in creative and reflective activities that support engagement with narrative situations through activity. This mixed-methods study examined whether participation in T. A. L. E. was associated with gains in emotion comprehension in children aged 7–9 years (*N* = 197; intervention = 99, control = 98; 97 boys, 100 girls) from five elementary schools in Budapest. The intervention was conducted in 2023 and 2024. Quantitative outcomes were assessed with the Test of Emotion Comprehension before and after the 12-session intervention, with age, gender, and cognitive ability included as control variables, while semi-structured interviews captured children’s social–emotional engagement. Results indicated that children in the intervention group showed larger gains than those in the control group, with a substantial effect size in this researcher-led implementation context (*η_p_^2^* = 0.32), suggesting that narrative participation may provide a developmentally meaningful context for emotional understanding. Qualitative findings suggested greater emphasis on self-reflection and perspective-taking among children showing larger gains. Teacher and psychology student evaluations provided preliminary acceptability evidence. These findings support a view of social–emotional learning not only as the acquisition of competencies, but as a process of meaning-making emerging through participation in socially and emotionally meaningful experiences, as examined here within narrative-based classroom contexts.

## 1 Introduction

Social and emotional learning (SEL) has emerged as a prominent focus in contemporary education, referring to the development of core competencies – such as self-awareness, self-management, social awareness, relationship skills, and responsible decision-making – that support effective functioning in social and emotional contexts (Collaborative for Academic, Social, and Emotional Learning, 2020). Early emotional development also plays a foundational role in children’s school readiness and later academic and social adjustment (Raver, 2002). Within this framework, emotion comprehension constitutes a key developmental component, reflecting children’s increasing ability to understand the causes, consequences, and regulation of emotions in themselves and others (Pons et al., 2004; Pons and Harris, 2019). Early elementary school represents a critical period for this development, as children begin to integrate emotional experiences with increasingly reflective forms of understanding through participation in socially meaningful activities.

One important context for such development is narrative engagement, which provides structured yet flexible opportunities for encountering emotionally meaningful situations, engaging with multiple perspectives, and reflecting on social experiences in socially embedded contexts. Through storytelling and subsequent interactional activities, children engage with characters’ intentions, conflicts, and outcomes, supporting the gradual transition from intuitive to more explicit and reflective forms of emotion understanding (Bruner, 1990; Nelson, 2007; Nicolopoulou, 1997; Hibbin, 2016). Rather than functioning merely as a vehicle for conveying content, narrative engagement may be understood as a participatory process through which children actively construct meaning about emotional and social situations.

From this perspective, development is shaped not only by exposure to emotionally relevant material, but also by how children engage with such material through activity, interaction, and reflection. These processes are particularly relevant in early school contexts, where structured opportunities for guided reflection remain limited in standard instructional approaches.

During this period, development arises not only from acquiring emotion knowledge but also from participating in socially embedded, child-centered narrative activities that provide contexts for exploring emotions, intentions, and social perspectives. These socially embedded experiences are thought to support gradual representational change, whereby children move from intuitive responses toward increasingly reflective understanding of emotions (Fonagy et al., 2002; Nelson, 2007; Overton, 2015). Storytelling allows children to encounter emotionally salient situations, fostering intuitive perspective-taking and early self-reflection, linking emotional understanding with social cognition (Bruner, 1990; Nicolopoulou, 1997; Piaget, 1962; Rogoff, 2003; Vygotsky, 1978). Early forms of perspective-taking often emerge as intuitive, non-verbal sensitivity to others’ emotions, which can be understood as a developmental precursor to more explicit and reflective forms of emotion understanding (Nelson, 2007). Within developmental frameworks, this progression supports the transition from implicit to increasingly articulated representations of emotion (Fonagy et al., 2002; Pons et al., 2004).

School-based narrative and dialogic interventions have supported emotion comprehension through guided emotion talk, structured discussion, dialogic reading, and perspective-taking (e.g., Grazzani et al., 2016; Ornaghi et al., 2014; Tenenbaum et al., 2008). These approaches consistently highlight the role of shared reflection and socially mediated meaning-making in supporting emotional development. However, less is known about how orally mediated narrative engagement – particularly when grounded in culturally diverse folktales – may support emotion comprehension through children’s reflective participation. In particular, there is limited understanding of how such engagement functions as a repeated classroom process, and how children’s active, experience-based participation relates to developmental change in emotion comprehension. Beyond these approaches, several European SEL programs have also demonstrated effectiveness in promoting broader social-emotional competencies in school contexts (e.g., Cefai et al., 2014; Simões et al., 2021; Sklad et al., 2012).

While sharing common ground with dialogic read-aloud and discussion-based approaches, the present approach examines orally mediated folktale storytelling as a repeated classroom process involving ritualized narrative engagement, creative activity, and reflection across sessions. Rather than treating oral storytelling as instructional content, the study conceptualizes it as a classroom context for activity-mediated engagement with narrative situations, in contrast to dialogic approaches where meaning is made explicit through guided conversation. In the present approach, reflection is primarily mediated through activity rather than direct interpretation.

In the present study, reflective engagement is conceptualized as children’s active involvement in meaning-making processes emerging through narrative participation. While sessions include brief, experience-based SEL activities (e.g., emotion-focused check-ins such as “What color is your day?”, breathing-based regulation exercises, and strength-based prompts such as “What is your superpower?”), these activities function as orienting conditions that prepare children for engagement with narrative situations by supporting emotional awareness, attention, and regulatory readiness, thereby shaping how children engage with subsequent narrative experiences. As such, they are not treated as independent instructional components, but as preparatory elements that shape the quality of subsequent narrative participation. Rather than engaging with narratives primarily through direct interpretation, children engage with the narratives indirectly through creative, multimodal activities that allow personal and social meanings to emerge, and these are subsequently elaborated through guided reflection in a socially shared context. Reflective engagement includes processes such as perspective-taking, linking narrative situations to personal and social experience, and considering alternative viewpoints. It is treated as an interpretive construct derived from children’s accounts, rather than as a directly measured psychometric variable.

The T. A. L. E. program (Tale Activates Learning and Emotional-Growth) was developed to address this gap by integrating oral storytelling, guided dialogue, and brief reflective activities within a structured classroom format. While T. A. L. E. includes brief explicit SEL activities, it does not rely primarily on direct instruction; instead, it emphasizes activity-mediated engagement, in which children participate in emotionally salient narrative situations that invite perspective-taking and reflective interpretation. In this sense, the program conceptualizes social–emotional learning as emerging through participation in meaningful social activity, aligning with sociocultural and developmental perspectives (Bronfenbrenner and Morris, 2007; Rogoff, 2003; Vygotsky, 1978). T. A. L. E. draws on five core social–emotional competencies – self-awareness, self-management, social awareness, relationship skills, and responsible decision-making – to structure its activities in alignment with established SEL frameworks (Collaborative for Academic, Social, and Emotional Learning, 2020).

Meta-analytic evidence indicates that universal, school-based SEL programs enhance social–emotional competencies, prosocial behavior, and academic outcomes (Cipriano et al., 2023; Shi et al., 2025; Zhao and Sang, 2025). Within this framework, Kernel exercises – brief, structured activities integrated into narrative sessions – support attention, emotion regulation, and reflective engagement in developmentally appropriate ways (Embry and Biglan, 2008; Dweck, 2006; Immordino-Yang & Damasio, 2007). These elements may support reflective and regulatory processes relevant to emotion comprehension, linking brief structured activities with children’s subsequent engagement in narrative situations. The intervention draws on oral storytelling traditions that remain educationally salient in the Hungarian context.

Narrative activities have been linked to young children’s social–emotional development (Gunawardena and Koivula, 2024; Nelson, 2007; Nicolopoulou et al., 2015). However, the role of oral storytelling within evidence-based SEL interventions targeting emotion comprehension remains insufficiently examined, particularly at the process level. This leaves limited understanding of how culturally varied storytelling can foster emotion comprehension and broader SEL development across contexts (Macheta et al., 2023; Pons and Harris, 2019). Age, gender, and cognitive ability may influence children’s responses to intervention, although prior evidence in early elementary school is mixed (Chaplin and Aldao, 2013; Denham et al., 2012; Izard et al., 2001; Pons et al., 2004).

### 1.1 The present study

To address this gap, the present study adopts a developmental, process-oriented perspective to examine how socially embedded narrative engagement functions within classroom contexts in supporting the development of emotion comprehension in early elementary school, a period marked by rapid developmental change in emotional and social understanding.

Using a mixed-methods design, we investigate whether participation in the T. A. L. E. intervention is associated with gains in emotion comprehension, while also exploring how children describe their engagement with narrative-based activities in terms of perspective-taking and reflective social–emotional insights. Age, gender, and fluid reasoning (i.e., the ability to solve novel problems independent of prior knowledge; Cattell, 1963) are included as control variables in the primary analyses and examined as potential moderators in exploratory analyses.

By integrating quantitative and qualitative data, the study aims not only to assess whether developmental change occurs, but also to illuminate the reflective and relational processes that may be relevant for interpreting such change in classroom contexts. In doing so, the study contributes to a more process-oriented understanding of how narrative-based participation may function as a developmentally meaningful context linking emotional experience, social interaction, and emerging reflective understanding within the broader framework of social–emotional learning.

The study addressed three related questions: (1) whether participation in T. A. L. E. was associated with greater gains in emotion comprehension than in a control group; (2) whether age, gender, and fluid intelligence functioned as potential moderators; and (3) whether improvement in emotion comprehension was reflected in children’s qualitative accounts of reflective social–emotional engagement.

*H1*: The intervention group was expected to show greater gains in emotion comprehension than the control group (Cipriano et al., 2023; Grazzani et al., 2016; Ornaghi et al., 2014; Shi et al., 2025).

Exploratory hypotheses were as follows:

*H2*: Intervention gains may vary as a function of age, given developmental differences in emotion understanding (Pons et al., 2004; Denham et al., 2012).

*H3*: Intervention gains may vary as a function of fluid intelligence, given possible links between reasoning and emotion comprehension, although prior evidence is mixed (Izard et al., 2001; Pons et al., 2004).

*H4*: Intervention gains were not expected to differ as a function of gender (Chaplin and Aldao, 2013; Izard et al., 2001).

## 2 Materials and methods

### 2.1 Participants

A total of 208 children from ten second-grade classrooms across five elementary schools in Budapest (representing three districts) were recruited following an initial pilot phase conducted in autumn 2022. The main intervention and data collection were conducted during the 2023 and 2024 school terms. After accounting for absences and attrition (5.3%, *N* = 11), complete pre- and post-intervention quantitative data were available for 197 children. Attrition was low and did not appear to affect comparability between intervention and control groups. The final analytic sample included 99 children in the intervention group and 98 in the control group, predominantly Hungarian-speaking, with a few Roma, Turkish, and Ukrainian children.

Participants were aged 7–9 years (M = 7.96, SD = 0.54), with a balanced gender distribution (97 boys, 49.2%; 100 girls, 50.8%). No significant differences were observed between the intervention and control groups regarding age, t (195) = 0.73, *p* = 0.464, or gender, χ^2^(1) = 0.13, *p* = 0.721, *φ* = −0.03, supporting baseline comparability. The sample was relatively homogeneous in terms of ethnicity and socioeconomic status, consisting mainly of middle-class, Hungarian-speaking children; other sociodemographic variables were not systematically collected.

Schools participated following principal approval, and classrooms were randomly assigned to intervention or control conditions, preserving peer and instructional structures within intact classrooms. Control classrooms continued standard curricular activities. Children with significant sensory or cognitive impairments attended the T. A. L. E. sessions but were excluded from standardized testing, as such impairments would preclude valid assessment of narrative-based outcomes. Of the intervention group, 99 children additionally contributed to the qualitative phase, providing data through semi-structured interviews and a child self-report questionnaire.

### 2.2 Study design

The present study employed a mixed-methods, cluster-randomized pretest–posttest design within natural classroom settings to examine the association between a narrative-based, dialogic social–emotional learning intervention and developmental change in emotion comprehension in early elementary children. Quantitative (TEC, CPM) and qualitative (semi-structured interviews) data were collected concurrently and analyzed separately, then interpreted together using a convergent design to provide a comprehensive, interpretive understanding of developmental changes in emotion comprehension.

The primary aim was to examine whether participation in the T. A. L. E. intervention, which combines oral storytelling, guided dialogue, and brief reflective exercises, was associated with gains in emotion comprehension in a developmentally sensitive classroom context. The design prioritized ecological validity and process-level interpretability, rather than large-scale implementation or routine teacher-led delivery. Schools were recruited via convenience sampling with principal consent, reflecting common constraints in school-based developmental research. Classes were randomly assigned to intervention or control conditions using a simple random lottery procedure.

Ethical approval was obtained from the institutional Research Ethics Committee, and all data were anonymized in accordance with GDPR (General Data Protection Regulation) and institutional guidelines. All procedures adhered to ethical standards for research with young children, minimizing stress during data collection. The mixed-methods approach captured both measurable developmental change and experiential patterns associated with these changes. Accordingly, findings are interpreted as evidence of association under controlled conditions rather than as definitive causal effects.

### 2.3 Intervention: the T. A. L. E. program

The T. A. L. E. intervention was a universal, classroom-based SEL program. It was implemented across autumn 2023 and spring 2024, with each classroom participating in one semester-long 12-session cycle during regular school hours, and attendance was monitored throughout implementation. Sessions lasted 45 min and were led by the first author, supported by up to four trained psychology students in non-instructional roles. These assistants supported session logistics (e.g., materials), observed group processes, and participated in pre- and post-session briefings. This researcher-led delivery model was chosen to maximize implementation fidelity during initial developmental testing, while acknowledging potential facilitator-related influences.

Implementation was supported by a detailed manual (100 + pages), including session protocols, activity instructions, learning objectives, material lists, reflection prompts, and fidelity-support elements, which provides a basis for future teacher-led implementation. As part of implementation support, psychology students completed reflective observation journals after each session documenting session structure, materials used, child engagement, practical adaptations, and reflective observations. These journals informed implementation monitoring and post-session reflection, rather than serving as outcome measures.

In addition, two psychology students per semester participated in a separate research practicum and were involved only in administering standardized assessments and conducting semi-structured interviews.

All classroom teachers (*N* = 5) observed the sessions and were introduced to embedded Kernel exercises for optional classroom use. Kernel exercises are brief, evidence-based techniques targeting specific psychological processes (Embry and Biglan, 2008). Although the program draws on oral storytelling traditions, the present study conceptualizes T.A.L.E. primarily as a universal, classroom-based SEL intervention rather than as a therapeutic storytelling model.

#### 2.3.1 Session structure and narrative engagement process

Children participated in 12 sessions, each following a six-part structure: (1) ritual opening, (2) preparatory SEL activities, (3) oral storytelling, (4) creative processing, (5) guided reflection, and (6) ritual closing, with optional take-home activities supporting continuity beyond the sessions.

**Figure 1:**
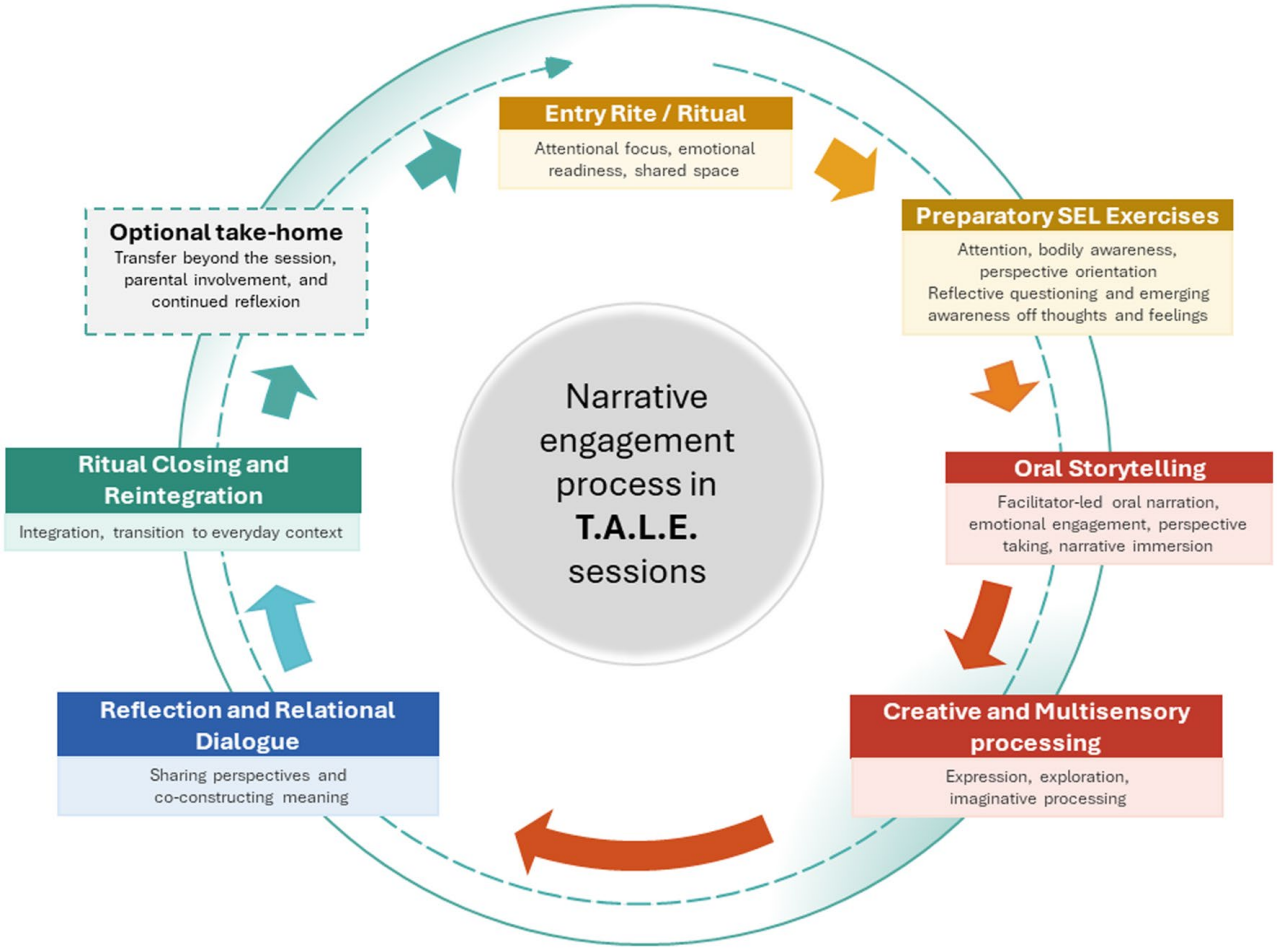
Process-oriented model of narrative engagement in the T. A. L. E. program. The figure illustrates the recurring phases of T. A. L. E. sessions, including ritual opening, preparatory SEL exercises, oral storytelling, creative processing, dialogic reflection, and closing. Each phase is associated with emotional, cognitive, and social forms of engagement. The model provides a process-oriented heuristic framework for understanding how narrative engagement may unfold through recurring, potentially spiraling processes across sessions and relate to children’s reflective and social–emotional processes, without implying causal relationships between components. It is adapted from the companion qualitative study.

The session structure and associated process components are illustrated in Figure 1, which provides an interpretive representation of how narrative engagement unfolds across phases of the intervention. The sequence moves from orientation (ritual opening and preparatory activities), through narrative immersion (storytelling), to multimodal processing and guided reflection, followed by emotional integration during closing.

Ritual opening and preparatory SEL activities introduced and experientially engaged children with the focal social–emotional themes of each session, providing a thematic and experiential foundation for subsequent narrative participation. These preparatory activities included brief emotion-focused prompts, breathing-based regulation exercises, and strength-based reflections designed to orient children toward emotional and social themes prior to storytelling.

Storytelling created a shared symbolic context for emotional and social meaning-making. This was followed by creative processing activities, through which children engaged with narrative experiences in embodied and expressive ways, mediating connections between story content and their own lived experiences. These experiences were subsequently elaborated through guided reflection in a socially shared, dialogic context, supporting perspective-taking and the articulation of personal meaning. Ritual closing provided emotional containment and facilitated transition back to the classroom context.

Emotion regulation was supported through the *Horseshoe Breathing* Kernel exercise. These brief, evidence-based activities (cf. Embry and Biglan, 2008) were embedded within narrative sessions to activate emotional awareness and regulation in developmentally appropriate ways. The exercise was linked to the Hungarian folktale *The Magic Bridle* and grounded in Brain House theory (Siegel and Bryson, 2011), a child-friendly model of brain functioning that helps children understand the relationship between emotional arousal, regulation, and reflective control. Within this framework, Horseshoe Breathing supported children in regulating emotional arousal prior to responding, thereby facilitating reflective control.

Perspective-taking was fostered through the ‘walking in another’s shoes’ activity, linked to the Bahamian folktale *The Price of the Most Loving Heart.* Children were invited to reflect on situations involving helping others and to consider how they might act in similar contexts.

#### 2.3.2 Folktale selection and pedagogical approach

Tales were selected to align with widely recognized SEL competencies (CASEL) and the developmental capacities characteristic of children aged 7–9 years, drawing on diverse cultural traditions (see Supplementary Table S1). Narrative immersion combined with guided reflection supported perspective-taking and early reflective processes (Hirsh-Pasek et al., 2015; Nicolopoulou, 2011; Reese et al., 2010). Ritualized openings and closings functioned to scaffold emotional engagement and to provide structured transitions into and out of reflective narrative space (Boldizsár, 2025).

Embedded Kernel exercises and growth mindset principles provided repeated, developmentally appropriate opportunities for practicing emotion regulation, attentional control, and reflective thinking within the narrative context (Embry and Biglan, 2008; Dweck, 2006). Classroom teachers observed the sessions and were introduced to these embedded practices, which could be flexibly incorporated into other classroom activities, thereby supporting continuity and the potential generalization of social–emotional learning beyond the intervention.

### 2.4 Instruments

#### 2.4.1 Quantitative measures

Test of Emotion Comprehension (TEC; Pons and Harris, 2000).

The TEC assesses nine components of emotion comprehension: emotion recognition, external causes, belief-based emotions, desire-based emotions, reminders, emotion regulation, concealment, mixed emotions, and moral emotions. The measure is grounded in a hierarchical developmental model, reflecting increasing complexity in children’s understanding of emotional processes across age (Pons et al., 2004; Pons and Harris, 2019). Widely used in developmental research, the TEC demonstrates strong construct and developmental validity across early- and middle-childhood samples, including research linking emotion comprehension and perspective-taking (e.g., Macheta et al., 2023; Ornaghi et al., 2014). Although the TEC is based on a developmental model of emotion comprehension that does not fully overlap with SEL frameworks, it captures key aspects of emotional understanding. The Hungarian adaptation showed robust psychometric properties and confirmed its cross-cultural applicability, while also highlighting minor variations in the ordering of specific components (Nemet et al., 2025). Total TEC scores (range: 0–9) were used as the primary outcome variable, with higher scores indicating more advanced emotion comprehension. The TEC test was administered pre- and post-intervention via a child-friendly computer-based version (*N* = 197).

Raven’s Colored Progressive Matrices (CPM; Raven and Court, 1962):

The CPM is a nonverbal measure of fluid intelligence, consisting of 36 items organized into three sets of increasing difficulty. Raw CPM scores were used as an index of fluid reasoning, conceptualized within the fluid–crystallized intelligence framework (Cattell, 1963). The Hungarian adaptation was used (Rózsa et al., 2006).
CPM scores were included as a covariate to control for individual differences in fluid reasoning.
Teacher Intervention Acceptability Profile (adapted from: Fudge et al., 2008).
Teachers completed a questionnaire assessing feasibility, perceived usefulness, and observed developmental relevance of T. A. L. E. sessions.
Children’s Self-Report Measure (see Supplementary Appendix S2).
A post-intervention emoji-based 3-point Likert scale assessed enjoyment, perceived emotional learning, social understanding, emotion regulation, and problem-solving. Children responded using visual anchors (sad, neutral, happy faces), which were coded as −1 (negative), 0 (neutral), and +1 (positive) for analysis. Data were interpreted at a descriptive level to complement quantitative findings.
The Strengths and Difficulties Questionnaire (SDQ; Goodman, 1997) was also administered. Due to missing or inconsistent responses, these data were not included in the present analyses.

#### 2.4.2 Qualitative measures

Semi-structured child interviews (*n* = 99) focused on experiences, preferred stories, and perceived emotional and social learning (see Supplementary Appendix S1). Open-ended, experience-focused questions captured emotional awareness, perspective-taking, and peer relationships. An inductive-deductive thematic analysis (Braun and Clarke, 2006) mapped emergent patterns onto CASEL SEL competencies. The analysis focused on identifying patterns of reflective engagement as expressed in children’s accounts of narrative participation, including how they connected story experiences to their own emotional and social worlds. Themes were rated on a 0–2 ordinal scale developed for the purposes of the study to capture variation in the depth of reflective engagement. These ratings were later compared descriptively across subgroups differing in TEC improvement.

### 2.5 Procedure

In autumn 2022, a single-session pilot was conducted with first grade classrooms to familiarize children and teachers with the T. A. L. E. format and procedures. Children provided verbal assent, and parents provided written consent; these children later participated in the main study as second graders, with full parental consent obtained. Children whose parents did not consent attended sessions but were excluded from standardized assessments. Attendance was monitored throughout the intervention.

Pre-intervention assessments were conducted approximately four weeks prior to the first intervention session. Post-intervention interviews and standardized assessments were conducted by the research team. To reduce demand characteristics, research assistants who had not been involved in intervention delivery participated in the assessment process. Children completed the TEC and CPM in familiar classroom settings during scheduled afternoon sessions (1–3 p.m.). Oral instructions and practice items ensured comprehension across the age range. Research assistants provided guided support under supervision to maintain developmental appropriateness.

Post-intervention assessments included the TEC, child self-report measures, and teacher acceptability profiles. All assessments were conducted in familiar classroom environments, with procedures adapted to ensure suitability for children aged 7–9 years.

As a courtesy, children in control-group classrooms later received a one-hour narrative-based session during regular class time.

### 2.6 Data analysis

Quantitative analyses examined changes in emotion comprehension over time and between groups, controlling for age, gender, and fluid intelligence. Qualitative interviews explored children’s self-reported experiences and reflective engagement, providing interpretive context for the quantitative findings.

#### 2.6.1 Quantitative analysis

TEC scores were normally distributed pre- and post-intervention (pre: skew = −0.27, kurtosis = 0.03; post: skew = −0.63, kurtosis = −0.22), supporting the use of parametric tests. Baseline equivalence was assessed with independent t-tests.

To test the primary hypothesis (H1), a Linear Mixed Model (LMM) was employed. The model specified TEC scores as the outcome variable, with Time (pre vs. post) and Group (intervention vs. control) as fixed factors, including their interaction term (Time × Group). To control for individual differences, gender was included as a categorical predictor, while age and fluid intelligence (CPM) were included as fixed continuous covariates. Age and CPM scores were mean-centered prior to analysis to ensure interpretability. Fixed effects were tested using Type III tests. To account for the hierarchical structure of the data and repeated measurements, Subject ID and Classroom ID were specified as random effects (random intercepts). Effect sizes for fixed effects were reported as partial eta-squared (*η_p_^2^*) to capture practical developmental significance (small = 0.01, medium = 0.06, large = 0.14; Cohen, 1988; Ellis, 2010). Given the educational context and high-fidelity implementation conditions, effect sizes were emphasized to reflect developmental relevance beyond statistical significance to capture the practical magnitude of change. Random effects were assessed using Wald tests and supplemented by the Intraclass Correlation Coefficient (ICC). Significant Time × Group interactions were followed up with simple effect analyses, reporting Estimated Marginal Means (EMM) and Standard Errors (SE) for both groups at each time point, calculated at the mean values of Age and CPM. To fully decompose the interaction, we performed both within-group comparisons (pre-intervention vs. post-intervention) and between-group comparisons (control vs. intervention at each time point). To account for multiple testing, *p*-values were adjusted using the Sidak correction. Effect sizes for these simple effects were reported as Cohen’s *d*.

To address the exploratory hypotheses (H2–H4), a second, more complex LMM was constructed. This model included the three-way interactions of Time × Group × Age, Time × Group × Gender, and Time × Group × CPM, along with all necessary lower-order two-way interactions and main effects to maintain model hierarchy. This complex model was compared against the initial parsimonious model using the Akaike Information Criterion (AIC) and Bayesian Information Criterion (BIC). Lower values in these criteria were used to identify the more parsimonious model with superior fit, guiding the final selection of the analysis framework.

Analyses were not preregistered. Analyses were performed using JASP (Version 0.96.0), an open-source statistical software environment that operates as a graphical user interface for R.

#### 2.6.2 Qualitative analysis

Qualitative analyses focused on identifying patterns of reflective engagement related to emotion comprehension. Semi-structured interviews (*n* = 99) were analyzed thematically (Braun and Clarke, 2006). An inductive-deductive approach allowed themes to emerge from children’s narratives while being conceptually anchored in CASEL’s SEL competencies. Coding was conducted in multiple stages: the lead researcher and a co-coder independently coded half of the transcripts, resolving ambiguities through discussion; a qualitative expert reviewed the process. Codes were organized into broader themes and subthemes. All transcripts were then re-coded using this finalized coding structure, and frequencies of coded instances were compiled in Excel. Using the 0–2 ordinal rating system described above, summed intensity scores were calculated as descriptive indicators of variation in the depth of reflective engagement. The scale functioned as a descriptive coding aid to characterize relative elaboration in children’s responses, rather than as a formal psychometric measure. To explore possible correspondence between qualitative engagement patterns and quantitative outcomes, participants were grouped for descriptive purposes only according to improvement in total TEC scores (average or below-average vs. above-average improvement, based on the sample mean change), and theme frequencies and intensity scores were compared descriptively across these groups. This grouping was used for non-inferential comparison only; no inferential statistical tests were conducted, and the groups do not represent distinct developmental categories. Inter-rater reliability was assessed by training an external coder with the finalized codebook and ordinal system. The external coder independently coded all transcripts, and codes were compared, yielding high inter-rater agreement for thematic presence (Cohen’s *κ* = 0.868).

#### 2.6.3 Integration of quantitative and qualitative data

Quantitative and qualitative findings were integrated at the interpretive level within a convergent, theory-driven mixed-methods design. Quantitative and qualitative data were collected concurrently, analyzed separately, and then interpreted together. Quantitative results provided evidence of change in emotion comprehension, while qualitative data contextualized these changes by examining whether patterns of reflective engagement varied descriptively across groups differing in TEC improvement. This integration was intended to explore possible process-level convergence rather than test formal associations. Teacher and child reports provided supplementary contextual insight.

## 3 Results

### 3.1 Baseline comparisons

Independent samples *t*-tests confirmed no significant differences between intervention and control groups in TEC scores (*t* (195) = −0.80, *p* = 0.423, *d* = −0.12) or Raven’s CPM scores (*t* (195) = 1.69, *p* = 0.093, *d* = 0.24), supporting baseline equivalence.

### 3.2 Main quantitative results

#### 3.2.1 Primary intervention effects on emotion comprehension

To test the primary hypothesis (H1), a Linear Mixed Model (LMM) was conducted on TEC scores. Analysis of the random effects structure revealed that the Subject ID random intercept was marginally significant (Wald *Z* = 1.91, *p* = 0.056), accounting for 14% of the total variance (ICC = 0.14). In contrast, the Classroom ID random intercept was not significant (Wald *Z* = 0.87, *p* = 0.386), explaining only 2% of the variance (ICC = 0.02). This suggests limited detectable classroom-level variance in baseline scores, although cluster-level influences cannot be fully excluded. Regarding the fixed effects (see Table 1), a highly significant Time × Group interaction was observed, *F* (1, 195.00) = 90.43, *p* < 0.001, with a substantial effect size (*η_p_^2^* = 0.32). While the main effects of Group and Time were also statistically significant, they were not interpreted independently due to the presence of a robust interaction. Among the control variables, fluid intelligence (CPM) was significantly associated with higher TEC scores, while age showed a marginal trend, and the effect of gender was not significant. To decompose the significant interaction, a simple effects analysis was performed. Table 2 presents within-group changes; between-group comparisons are reported in the text. The control group showed no significant change in emotion comprehension over time (ΔEMM = −0.30, *SE* = 0.17, *p* = 0.080, Cohen’s *d* = −0.20). In contrast, the intervention group exhibited a marked and significant increase in TEC scores (ΔEMM = 1.96, *SE* = 0.17, *p* < 0.001, d = 1.32). Furthermore, while there was no significant difference between the two groups at baseline (*p* = 0.441, *d* = 0.11), the intervention group significantly outperformed the control group at the post-intervention assessment (*p* < 0.001, *d* = 1.60). These findings provide strong support for H1, indicating that participation in the T. A. L. E. program was associated with intervention-related gains in emotion comprehension.

**Table 1:** Fixed effects results for the primary intervention linear mixed model (H1) on TEC scores.

Predictors	*df1*	*df2*	*F*-	*p*	*η_p_^2^*
Group	1	8.41	50.58	**< 0.001**	—
Time	1	195.00	49.20	**< 0.001**	0.20
Gender	1	191.41	2.62	0.107	0.01
Age	1	150.60	3.28	0.072	0.02
CPM	1	185.23	5.07	**0.025**	0.03
Time × Group	1	195.00	90.43	**< 0.001**	0.32

TEC = Test of Emotion Comprehension; CPM = Colored Progressive Matrices. Results are based on a Linear Mixed Model (LMM) with Subject ID and Classroom ID included as random intercepts to account for the nested data structure. Fixed effects were tested using Type III Wald tests; *df2* was calculated using the Satterthwaite approximation. Main effects for Group and Time represent marginal effects averaged across levels; given the robust Time × Group interaction, these should be interpreted in the context of the subsequent simple effects analysis. Effect sizes (*η_p_^2^*) represent partial eta-squared; however, values are not reported for Group because the Group effect is tested at the classroom-level (between-cluster), resulting in limited degrees of freedom that may yield unstable or biased effect size estimates. Continuous covariates were mean-centered. Bold values indicate significance at *p* < 0.05.

**Table 2:** Estimated marginal means for TEC scores pre- and post-intervention in the intervention and control groups.

Groups	Pre-intervention		Post-intervention		Difference				
	EMM	SE	EMM	SE	Δ EMM	SE	CI	*p*	*d*
Control	5.76	0.15	5.46	0.15	−0.30	0.17	[−0.63; 0.04]	0.080	−0.20
Intervention	5.93	0.15	7.89	0.15	1.96	0.17	[1.63; 2.29]	**< 0.001**	1.32

EMM = Estimated Marginal Means; SE = Standard Error; CI = 95% Confidence Interval for the difference; d = Cohen’s d. Results are derived from the simple effects analysis following the significant Time × Group interaction in the Linear Mixed Model. All estimates are evaluated at the mean values of the continuous covariates (age and CPM). *p*-values refer to the within-group change over time (post vs. pre). Sidak adjustment was applied for multiple comparisons. Bold values indicate significance at *p* < 0.05.

#### 3.2.2 Exploratory moderation analyses (H2–H4)

To investigate whether the intervention effect was moderated by demographic or cognitive factors, a more complex LMM was constructed including the relevant lower-order terms and the three-way interactions (Time × Group × Gender; Time × Group × Age; Time × Group × CPM). Model comparison indices favored the more parsimonious H1 model (AIC = 1326.04; BIC = 1337.92) over the complex exploratory model (AIC = 1339.43; BIC = 1351.23). The substantial difference in these criteria (ΔAIC > 13) indicates that including these moderators did not improve model fit. Consistent with the model comparison, the Time × Group interaction remained robust and highly significant even in the complex model, *F*(1, 189) = 87.86, *p* < 0.001, *η_p_^2^* = 0.32, while none of the three-way interactions reached significance: Time × Group × Gender (*p* = 0.518, *η_p_^2^* < 0.01), Time × Group × Age (*p* = 0.808, *η_p_^2^* < 0.01), and Time × Group × CPM (*p* = 0.361, *η_p_^2^* < 0.01). We found no evidence in this sample that intervention effects differed as a function of age, gender, or fluid intelligence. Detailed results of the exploratory model are provided in Supplementary Table S2. Separate comparisons with Hungarian normative data are provided in Supplementary Table S6.

### 3.3 Thematic analysis of qualitative interviews

Thematic analysis followed Braun and Clarke (2006), using an inductive-deductive approach. Children’s responses were initially coded inductively, then mapped onto CASEL’s core social–emotional learning competencies (Collaborative for Academic, Social, and Emotional Learning, 2020). Themes were identified through iterative coding and clustering procedures described in the Methods section. Values represent mean frequencies and mean summed intensity scores per participant within each TEC improvement group. Intensity scores were based on summed ordinal ratings (0–2) across coded segments, reflecting the depth of children’s reflective engagement within each theme (see Supplementary Table S3 for the thematic coding structure and frequency/intensity summary).

Five overarching themes were identified. The most prominent themes were self-awareness and personal reflection (Theme 2) and social-relational reflections (Theme 3). At a descriptive level, Themes 2 and 3 appeared most closely aligned with the broader pattern of gains in emotion comprehension, whereas Theme 1 primarily reflected general enjoyment. Theme 5, although less frequent, also showed comparatively high summed intensity scores, suggesting that these reflections, when present, were often expressed in more elaborated forms, particularly in relation to problem-solving.

### 3.4 Integration of quantitative and qualitative findings

Children with larger gains tended to show stronger qualitative engagement in Themes 2 and 3, with somewhat clearer differences in Theme 3, suggesting descriptive alignment between reflective participation and emotion comprehension gains, including perspective-taking processes. Table 3 summarizes mean theme frequencies and mean theme intensity scores by TEC improvement group, with illustrative excerpts in Supplementary Table S4. These comparisons serve as heuristic contrasts to illustrate possible developmental alignment, not as formal tests of association or effect. These qualitative indicators provide important context: TEC improvements alone would be primarily interpretable at a psychometric level, with limited developmental context. Integration of quantitative and qualitative data suggests possible process-level convergence: narrative participation, together with creative multimodal engagement and guided reflection, may provide a meaningful context for perspective-taking and emotion comprehension. Formal testing of these active ingredients requires future component-based or mediation analyses, as the present design does not permit causal inference at the process level.

**Table 3:** Descriptive frequencies and intensity scores of children’s reflections by TEC improvement group.

	Average or below-average TEC improvement		Above-average TEC improvement	
	M	SD	M	SD
(a) Theme frequencies				
Theme 1: Positive experiences of storytelling	1.88	1.12	2.14	1.56
Theme 2: Self-awareness and personal reflection	2.81	1.66	3.14	3.24
Theme 3: Social-relational reflections	1.07	0.99	1.40	1.21
Theme 4: Creative expression	1.15	0.91	1.14	0.61
Theme 5: Responsible decision-making	0.46	0.81	0.53	0.94
(b) Theme intensity scores				
Theme 1: Positive experiences of storytelling	3.32	1.85	3.28	1.78
Theme 2: Self-awareness and personal reflection	2.95	1.96	3.02	2.45
Theme 3: Social-relational reflections	1.76	1.84	2.40	2.28
Theme 4: Creative expression	1.78	1.35	1.57	0.92
Theme 5: Responsible decision-making	0.93	1.54	0.91	1.71

TEC = Test of Emotion Comprehension. Groups represent children showing average or below-average versus above-average improvement in total TEC scores, based on mean score change. Frequency values represent mean numbers of coded instances per participant. Intensity scores represent mean summed ordinal ratings (0–2) reflecting the relative depth of reflective engagement. Comparisons are descriptive only and do not represent inferential group differences.

### 3.5 Teachers’ and students’ evaluations

Teacher and psychology student evaluations are summarized in Table 4. Elementary teacher observers (*N* = 5) and psychology student raters (*N* = 7) rated classroom applicability, satisfaction, behavioral and emotional impact, and developmental relevance of the T. A. L. E. sessions on a 6-point Likert scale (−3 to +3), with higher scores indicating more positive evaluations.

**Table 4:** Summary evaluation of the T. A. L. E. program by teachers and psychology students.

Evaluation criteria	Teacher M	Teacher SD	Student M	Student SD
Satisfaction with the method	2.40	0.97	2.64	0.63
Classroom applicability	2.40	0.97	2.50	0.52
Impact on behavior and emotion regulation	1.70	1.06	2.64	0.50
Support for classroom management	1.90	0.99	2.29	0.91
Applicability for diverse students	1.60	1.26	2.07	1.59
Lack of negative side effects	2.90	0.32	2.36	0.63

6-point Likert scale ranging from −3 (lowest) to +3 (highest); higher scores indicate more positive evaluations.

Teachers rated T. A. L. E. highly across all dimensions, including classroom applicability, satisfaction, and perceived developmental relevance. No negative side effects were reported. Slightly lower ratings for classroom management likely reflect the flexible, discussion-based format rather than limitations of the intervention itself.

Psychology students similarly reported high satisfaction and willingness to recommend T. A. L. E., with minor variations across dimensions. Neither group indicated any negative effects. Overall, teacher, student, and child feedback suggest that T. A. L. E. was positively received in the participating classrooms. Given the small number of teacher and student raters and the exploratory nature of these data, these evaluations should be interpreted as informative contextual ratings rather than as independent evidence of effectiveness. The questionnaire was adapted from Fudge et al. (2008).

These evaluations are best understood as non-inferential indicators of acceptability and perceived relevance, not as formal outcome measures.

### 3.6 Children’s self-reported experiences

Of the 99 children who participated in structured interviews, 93 completed a child-friendly, 3-point emoji Likert scale using visual anchors (sad, neutral, happy faces), coded as −1 (negative), 0 (neutral), and +1 (positive) (see Supplementary Appendix S2). The remaining six children were not available to complete the tool due to absence or scheduling conflicts during data collection sessions. Non-inferential results from the child self-report measure are presented in Supplementary Table S5, which summarizes mean item scores for each response domain, reflecting overall enjoyment, emotional understanding, social perspective-taking, emotion regulation, and problem-solving.

These self-reported experiences appear to conceptually align with qualitative interview findings, particularly Theme 2 (Self-awareness and personal reflection) and Theme 3 (Reflections on social and relational skills), offering convergent, process-level context for interpreting gains in emotion comprehension. Importantly, the evaluations were not analyzed by TEC improvement level and therefore cannot be interpreted as direct correlates of differential TEC gains. Rather, they reflect general subjective experiences of the intervention. Given its non-validated nature, the emoji Likert scale provides descriptive insights only and should not be interpreted inferentially, consistent with established methodological considerations regarding non-validated self-report measures. Overall, these exploratory findings suggest that T. A. L. E. was positively received by children.

### 3.7 Summary

Participation in T. A. L. E. was associated with improvements in emotion comprehension across the intervention period. Qualitative findings suggested that children engaged actively with narrative activities, particularly through activity-mediated reflection and references to social and emotional experiences. Teacher and child feedback further indicated that the program was well received and perceived as relevant within the classroom context. Overall, these findings support the developmental plausibility of T. A. L. E. and provide a foundation for future research examining teacher-led implementation, long-term outcomes, and broader dissemination.

## 4 Discussion

### 4.1 Interpretation of findings in light of developmental and SEL research

This study suggests that narrative-based classroom engagement may support the development of emotion comprehension in early elementary school, while pointing to reflective meaning-making as a potentially relevant process-level pathway through which these changes may be interpreted. In this context, brief preparatory SEL activities can be understood as orienting conditions that support children’s readiness for narrative engagement by shaping emotional awareness, attention, and regulatory processes prior to participation. Rather than functioning as isolated instruction, these brief activities appeared to prepare children for engaging with narrative situations in a more attentive and emotionally attuned way, thereby supporting the depth of subsequent engagement.

These findings support H1, indicating that participation in T. A. L. E. was associated with greater gains in emotion comprehension than in the control group. They also address the study’s third aim by providing interpretive context for these gains through patterns of reflective engagement. Observed gains were consistent with the intervention’s emphasis on activity-mediated reflection, supported by dialogic elements, and align with a process-based interpretation of narrative-based SEL. This interpretation is supported by the descriptive convergence of quantitative and qualitative findings (Section 3.4), where higher levels of reflective engagement aligned with greater gains in emotion comprehension. It is noteworthy that mean emotion comprehension scores in the control group decreased slightly over time, although this change was not statistically significant. Given no theoretical reason to expect deterioration, this pattern is interpreted cautiously and may reflect measurement variability, developmental heterogeneity, or classroom-level contextual factors rather than meaningful decline. Overall, T. A. L. E. appeared to engage children in repeated classroom interactions through which emotional understanding may be gradually shaped over time (Fonagy et al., 2002; Hibbin, 2016; Nelson, 2007).

Compared to many reading-based approaches, T. A. L. E. places greater emphasis on live oral storytelling, creative processing, and children’s indirect engagement with narrative situations, although modalities were not directly compared and future research should examine their relative contributions. Importantly, narrative meaning was not constructed through explicit discussion of story content, but emerged indirectly through children’s engagement in creative, embodied, and multimodal activities.

This approach complements structured, skills-based SEL programs by emphasizing experience-based developmental pathways that foster perspective-taking, self-reflection, and emotional understanding through participation and guided dialogue. Although both teachers and psychology students evaluated the program positively, minor differences emerged in areas such as classroom management and applicability, possibly reflecting practical implementation constraints.

In this sense, the conceptual model presented in Figure 1 offers a framework for understanding how recurring phases of narrative engagement may support the development of emotion comprehension in classroom contexts. It is not intended as a formal causal model, but as a theoretically grounded representation that organizes observed process patterns across repeated classroom interactions.

### 4.2 Process-level interpretation and developmental meaning-making

Rather than isolating discrete components, the results can be interpreted as a sequence in which brief explicit preparation supports children’s subsequent engagement with narrative situations through activity and reflection. Within this sequence, narrative participation appears to support emotion comprehension not through direct interpretation of story content, but through children’s active, embodied, and multimodal engagement with narrative situations.

Reflective processes may thus be understood as emerging within this engagement, rather than being externally imposed through explicit instruction. In this sense, these processes may be understood as recurrent interactions across sessions through which developmental change may gradually emerge. These activities supported children in connecting narrative content to lived experience, which was subsequently elaborated through guided reflection.

To further interpret these patterns, we examined process-level themes reflected in children’s accounts. These themes suggested early forms of perspective-taking and socially oriented reflection emerging around narrative situations (Nelson, 2007). As illustrated in Figure 1, such processes are embedded across recurring phases of the session structure rather than occurring as isolated instructional components. These phases corresponded particularly to qualitative themes of self-awareness (Theme 2) and social-relational reflection (Theme 3). Descriptively, self-awareness themes appeared more frequently, whereas social-relational reflections, though less frequent, showed higher mean intensity ratings, suggesting more elaborated forms of reflection. This pattern is consistent with developmental perspectives emphasizing recurrent and increasingly complex engagement.

Storytelling, guided dialogue, and embedded Kernel exercises may have supported the integration of self- and other-referential reflection, although their distinct contributions were not tested. Taken together, the findings suggest that narrative participation may support the emergence of emotion comprehension within classroom contexts. These patterns suggest that explicit preparation and indirect narrative engagement may function in combination, rather than as alternative approaches to social–emotional learning. Consistent with exploratory hypotheses H2–H4, no evidence emerged that intervention effects were moderated by age, gender, or cognitive ability.

### 4.3 Equity and culturally responsive SEL

T. A. L. E.’s use of culturally diverse narratives may provide a promising basis for future adaptation to more diverse classroom contexts. Although the sample in this study was relatively homogeneous in ethnicity and socioeconomic background, the core processes examined here – dialogic reflection, perspective-taking, and narrative engagement – may extend beyond the specific cultural context studied, although their expression is likely to vary across settings. Future implementation should explore teacher-led delivery in classrooms with greater cultural, linguistic, and socioeconomic diversity, examining how narrative-based SEL can be adapted and scaled in multiethnic educational settings. This would provide evidence regarding the generalizability of the processes emphasized in T. A. L. E., while also highlighting strategies for culturally responsive pedagogy. Integrating teachers more actively through training, coaching, and co-facilitation could enhance scalability, ecological validity, and sustainability while preserving high-fidelity engagement with narrative and dialogic components. Teacher-led adaptation in diverse classrooms would allow examination of whether these gains remain robust under routine educational conditions, not solely researcher-led implementation.

### 4.4 Pedagogical implications

T. A. L. E.’s modular, predictable structure supports developmental consistency while remaining responsive to children’s emerging capacities. Narrative participation may provide experience-near contexts in which self- and other-referential processes are coordinated, fostering social–emotional development through developmentally aligned processes rather than prescriptive instruction. Teachers can adapt T. A. L. E. practices flexibly, ensuring relevance across diverse cultural and linguistic classroom settings.

## 5 Conclusions and limitations

### 5.1 Methodological and measurement considerations

The classroom-randomized design in convenience-sampled schools enhanced ecological validity and developmental sensitivity, but limits strong causal inference, reflecting common constraints in school-based developmental research. Because assignment occurred at the classroom level, observations were not fully independent, and classroom-level processes may have contributed to observed effects despite limited baseline between-classroom variation. Findings should therefore be interpreted cautiously until replicated using analytic approaches that model clustering more directly.

Observed changes should be understood as shifts within ongoing developmental trajectories rather than stable end-state competencies. Intervention delivery by the first author and trained assistants prioritized implementation fidelity, but limits conclusions about teacher-led scalability. The relatively large effect size should therefore be interpreted within the context of researcher-led, high-fidelity implementation and a proximal outcome measure, with possible contributions from facilitator-related processes such as narrative delivery and relational attunement.

The study also lacked long-term follow-up, leaving open whether gains reflect transient or enduring change, and teacher and student feedback provided contextual support rather than independent causal evidence. The sample was relatively homogeneous, although the use of culturally diverse storytelling may support broader relevance.

Future research should examine implementation across more demographically diverse classrooms and teacher-led models to test the robustness of T. A. L. E.’s mechanisms. Because a detailed implementation manual has already been developed, future studies can examine teacher-led delivery through a training-and-coaching model supported by session protocols, fidelity indicators, and reflective observation journals to monitor implementation adaptations across classrooms.

### 5.2 Broader contribution and developmental implications

Taken together, the findings address the study objectives by linking intervention-related gains, exploratory moderation analyses, and qualitative accounts of reflective engagement within a coherent developmental interpretation. Oral storytelling provides experience-near contexts that support early perspective-taking and self-reflection. T. A. L. E.’s narrative and dialogic components appear to support processes relevant to emotion comprehension, consistent with socially grounded processes and gradual representational change, as reflected in the convergent pattern of quantitative and qualitative findings. This framework offers a basis for future work on classroom-based narrative SEL across diverse educational contexts. More broadly, the study contributes to the literature by advancing a process-oriented account of narrative engagement and providing preliminary empirical support for its relevance to emotion comprehension in early school settings.

Rather than positioning narrative as content to be interpreted, the present findings suggest that narrative-based participation may function as a repeated classroom context in which children engage with emotional and social situations through activity, interaction, and reflection. In this sense, social–emotional learning may be understood not only as the acquisition of competencies, but as a process of meaning-making emerging through repeated, socially embedded experiences.

## Data Availability

The datasets generated and analyzed in this study are available from the corresponding author upon reasonable request.
