# Supplementary material for: Narrative participation as a context for meaning-making in early school years: a mixed-methods study of emotion comprehension
Source: Front Psychol. 2026 Jun 3;17:1833673. doi: 10.3389/fpsyg.2026.1833673 (PMC13273900; doi:10.3389/fpsyg.2026.1833673)
Supplement: Supplementary file 1 [file Supplementary_file_1.docx]

**Narrative Participation as a Context for Meaning-Making in Early School Years: A Mixed-Methods Study of Emotion Comprehension**

**Supplementary Material**

**Appendix S1.** *Semi-Structured Interview Protocol*

The semi-structured interview protocol used in the present study was originally developed and implemented during the qualitative phase of this research (Német et al., unpublished manuscript). The interviews aimed to explore children's emotional, social, and cognitive engagement with the T.A.L.E. method through open-ended reflection. The interview included the following questions:

1. What was your overall experience with the T.A.L.E. method, and why?

2. Which folktale was your favourite, and why?

3. Which exercise did you like the most, and why?

4. Which creative activity (where we made something) did you enjoy the most, and why that one?

5. What will you take away from this experience? What did you learn?

**Appendix S2.** *T.A.L.E. Feedback Form (Child Self-Report Emoji Likert Scale)*

Please mark your answer with a checkmark (√). Respondent code:

| 1. I enjoyed the T.A.L.E. sessions. | 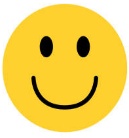 | 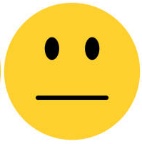 | 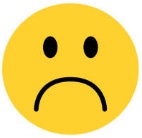 |
| --- | --- | --- | --- |
| 2. The T.A.L.E. sessions helped me get along better with my friends. | 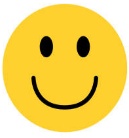 | 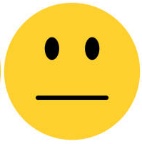 | 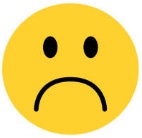 |
| 3. The T.A.L.E. sessions helped me understand how other people feel. | 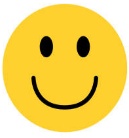 | 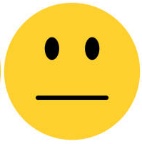 | 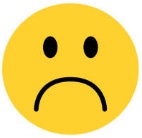 |
| 4. What I learned in T.A.L.E. helps me control my feelings. | 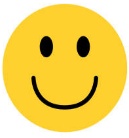 | 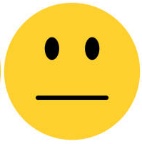 | 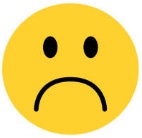 |
| 5. In T.A.L.E., I learned new words to describe feelings. | 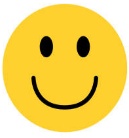 | 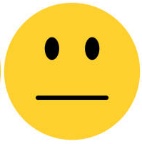 | 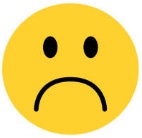 |
| 6. The story sessions helped me do better at school. | 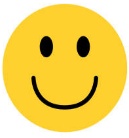 | 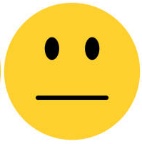 | 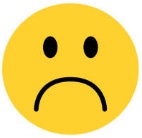 |
| 7. What I learned in T.A.L.E. helps me solve my problems better. | 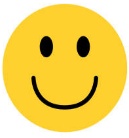 | 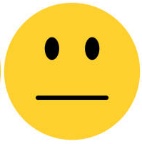 | 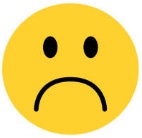 |

**2. Supplementary Tables**

**Table S1.** *Folktale Selection Aligned with CASEL Core SEL Competencies*

| **Core SEL Competency** | **Tales** |
| --- | --- |
| Self-awareness | *Where Do Stories Come From?* (Zulu folktale); *The Tea Master and the Samurai* (Japanese tale); *The Gingerbread Man* (American folktale) |
| Self-management | *The Magic Bridle* (Hungarian folktale);  *Sturdy Johnny and the Bread Hard as Stone* (Hungarian folktale) |
| Social awareness | *The Two Foxes* (Appalachian folktale);  *The Prize of the Most Loving Heart* (Guanima Island folktale) |
| Relationship skills | *The Stone and the Coachmen* (Romanian folktale); *Anansi and the Pot of Wisdom* (Ghanaian folktale) |
| Responsible decision-making | *The Brave Little Parrot* (Buddhist tale, told in Kamishibai format);  *The Twelve Months* (Greek folktale);  *How Did a Star Get Inside the Apple?* (Kamishibai tale) |

**Table S2.** *Fixed Effects Results of the Exploratory Linear Mixed Model (LMM) Testing for Potential Moderators (H2–H4) of Intervention Success on TEC Scores*

| **Predictors** | **df1** | **df2** | **F** | **p** | **η_p_²** |
| --- | --- | --- | --- | --- | --- |
| Group | 1 | 6.81 | 67.26 | < .001 | - |
| Time | 1 | 189.00 | 45.74 | <.001 | .19 |
| Gender | 1 | 189.00 | 2.55 | .112 | .01 |
| Age | 1 | 106.22 | 4.06 | .046 | .04 |
| CPM | 1 | 164.64 | 4.15 | .043 | .02 |
| Time × Group | 1 | 189.00 | 87.86 | <.001 | .32 |
| Time × Gender | 1 | 189.00 | 0.16 | .692 | < .01 |
| Time × Age | 1 | 189.00 | 0.41 | .521 | < .01 |
| Time × CPM | 1 | 189.00 | 0.51 | .476 | < .01 |
| Group × Gender | 1 | 189.00 | 0.03 | .868 | < .01 |
| Group × Age | 1 | 106.22 | 3.53 | .063 | .03 |
| Group × CPM | 1 | 164.64 | 0.45 | .502 | < .01 |
| Time × Group × Gender | 1 | 189.00 | 0.42 | .518 | < .01 |
| Time × Group × Age | 1 | 189.00 | 0.06 | .808 | < .01 |
| Time × Group × CPM | 1 | 189.00 | 0.84 | .361 | < .01 |

*Note*. TEC = Test of Emotion Comprehension; CPM = Colored Progressive Matrices. Results are based on a complex Linear Mixed Model (LMM) including three-way interactions to test moderating effects. Subject ID and Classroom ID were included as random intercepts. Fixed effects were tested using Type III Wald tests. The varying *df2* values reflect the hierarchical nature of the data: Group effects are tested at the cluster (classroom) level, while Time and its interactions are tested at the individual measurement level. Continuous covariates (Age, CPM) were mean-centered. Partial eta-squared is reported for all effects except the Group main effect, where cluster-level testing would lead to biased estimates. The Intraclass Correlation Coefficient (ICC) was .13 for Subject ID and .01 for Classroom ID. Bold values indicate significance at *p* < .05.

**Table S3.** *Aggregate Frequencies and Summed Intensity Scores for Thematic Codes*

| **Theme** | **Frequency** | **Intensity Score** |
| --- | --- | --- |
| 1. Positive experiences of oral storytelling-based sessions | 201 | 326 |
| 1.a. Learning experiences | 99 | 151 |
| 1.b. Impact of folktales on children's emotional well-being | 102 | 175 |
| 2. Self-awareness and personal reflection | 297 | 296 |
| 2.a. Emotion regulation in real-life situations | 72 | 135 |
| 2.b. Children's emotional skills | 118 | 161 |
| 2.c. Kernel exercises and self-reflection | 107 | 196 |
| 3. Reflections on social and relational skills | 125 | 211 |
| 3.a. Relational skills and community functioning | 103 | 175 |
| 3.b. Social awareness (empathy, understanding others) | 22 | 36 |
| 4. Experiences of creative expression | 113 | 164 |
| 5. Responsible decision-making | 50 | 91 |
| 5.a. Problem-solving | 38 | 64 |
| 5.b. Ethical considerations | 12 | 27 |

*Note.* Intensity scores reflect summed ordinal ratings across coded instances and may exceed frequency counts when instances receive higher-intensity ratings. Because excerpts could receive multiple subtheme codes, subtheme intensity scores are not additive and may exceed the intensity reported for the overarching theme.

**Table S4.** *Illustrative Interview Excerpts by TEC Improvement*

| Child code | Interview question & response | TEC improvement  category | TEC  Score  improvement | Coded Theme 2 | Coded Theme 3 |
| --- | --- | --- | --- | --- | --- |
| **Bac10** | **Which exercise was your favourite, and why?** “Horseshoe breathing. I tried it in the forest because my brother led me to a snake and I got scared and ran to my mother, then I used it and remembered it.” | Above average | 4 | 2a2, 2c2 | 3a2 |
| **Bac9** | **What do you take away, what did you learn?** “When the bunny and the other animals went to the eagle, I really liked that. Raisin meditation.” | Above average | 4 | 2b1, 2c2 | 3a1 |
| **Bac13** | **How was your overall experience with the T.A.L.E. program and why?** “It was good, we heard many stories, the sessions were exciting.” | Above average | 4 | 2b2 |  |
| **Bac17** | **Which exercise was your favourite, and why?** “Horseshoe breathing made me feel happier.” | Average-or-below-average | 1 | 2c2 | 0 |
| **Csb17** | **What do you take away, what did you learn?** “Nothing.” | Average-or-below-average | 0 | 0 | 0 |
| **Ka16** | **How was your overall experience with the T.A.L.E. program and why?** “Sometimes more tiring, sometimes helpful.” | Average-or-below-average | 0 | 2a2 | 0 |

*Note.* TEC = Test of Emotion Comprehension. Codes refer to subthemes within Theme 2 and Theme 3.

| **Table S5.** *Children’s Self-Reported Reflections on Emotional and Social Learning in T.A.L.E. Sessions (3-point Likert scale: −1 to 1)* | | | | | |
| --- | --- | --- | --- | --- | --- |
|  | | N | | M | |
| 1. I enjoyed the T.A.L.E. sessions. |  | 93 |  | .90 |  |
| 2. The T.A.L.E. sessions helped me get along better with my friends. |  | 93 |  | .73 |  |
| 3. The T.A.L.E. sessions helped me understand how other people feel. |  | 93 |  | .82 |  |
| 4. What I learned in T.A.L.E. helps me control my feelings. |  | 93 |  | .84 |  |
| 5. In T.A.L.E., I learned new words to describe feelings. |  | 93 |  | .87 |  |
| 6. The story sessions helped me do better at school. |  | 93 |  | .76 |  |
| 7. What I learned in T.A.L.E. helps me solve my problems better. |  | 93 |  | .82 |  |
| *Note.* 3-point emoji Likert scale ranging from −1 (negative) to +1 (positive). |  |  |  |  |  |

**Table S6.** *Comparison of Intervention and Control Group Scores with Hungarian Age-Based TEC Norms (Német et al., 2025)*

|  | Standard Hungarian scores | | | | Intervention | | | | | Control | | | | | |  |
| --- | --- | --- | --- | --- | --- | --- | --- | --- | --- | --- | --- | --- | --- | --- | --- | --- |
|  |  |  |  |  | Pre-intervention score | | Post-intervention score | |  | Pre-intervention score | | Post-intervention score | |  | | |
| Age | *M* | *LLCI* | *UCLI* | *SD* | *M* | *SD* | *M* | *SD* | ***ΔM*** | *M* | *SD* | *M* | *SD* | | ***ΔM*** | |
| 5 | 5.01 | 4.62 | 5.40 | 1.63 | 4.00 | — | 7.00 | — | 3.00 |  | — | — | — | | — | |
| 6 | 5.84 | 5.48 | 6.20 | 1.55 | 6.25 | 1.50 | 7.25 | 0.96 | 1.00 | 6.00 | 1.00 | 6.00 | 1.73 | | 0.00 | |
| 7 | 6.36 | 5.93 | 6.80 | 1.61 | 5.90* | 1.45 | 8.12** | 0.75 | 2.22** | 5.92* | 1.22 | 5.69** | 1.44 | | -0.23 | |
| 8 | 7.12 | 6.79 | 7.46 | 1.37 | 6.00** | 1.39 | 7.79** | 1.18 | 1.79 | 5.67** | 1.33 | 5.35** | 1.44 | | -0.32 | |
| 9 | 7.55 | 7.15 | 7.95 | 1.06 | 4.00 | — | 7.00 | — | 3.00 | 5.50 | 0.71 | 4.00 | 1.42 | | -1.50 | |

Note. “—” indicates data not available. * *p* < .05, ** *p* < .01. *Asterisks indicate significant deviations of group means from the corresponding Hungarian age-based normative mean.* *LLCI = lower limit of the 90% confidence interval; ULCI = upper limit of the 90% confidence interval*; ΔM = mean difference; TEC = Test of Emotion Comprehension.
